# Supplementary material for: Anemia prevalence and severity among pregnant refugee women settled in the West Nile region, Uganda
Source: PLoS One. 2025 Aug 22;20(8):e0329970. doi: 10.1371/journal.pone.0329970 (PMC12373188; doi:10.1371/journal.pone.0329970)
Supplement: Attachment — Submitted filename: PONE-D-24-37952_r2rsRN.docx [file pone.0329970.s003.docx]

PONE-D-24-37952

Anemia prevalence and severity among pregnant refugee women settled in the west Nile region, Uganda

PLOS ONE

Dear Dr. Bongomin,

Thank you for submitting your manuscript to PLOS ONE. After careful consideration, we feel that it has merit but does not fully meet PLOS ONE’s publication criteria as it currently stands. Therefore, we invite you to submit a revised version of the manuscript that addresses the points raised during the review process.

==============================

**ACADEMIC EDITOR:**

Study context and setting

- Provide more background on the refugee population (origin, duration in camps), healthcare structure, and the number of facilities serving refugees in Adjumani District.

Authors response: This has been updated

- Clarify the selection method for the three health facilities specify if it was random, purposive, or otherwise and how the sample size was distributed among them.

Authors response: This was random as indicated in the methods section and samples distributed proportionately.

Methods

- Explain if haematinics are routinely provided at ANC and whether their use was assessed.

Authors response: Yes, and this has been indicated. However, we didn’t assess this as was assumed to be universal. This is a limitation we have now acknowledged.

- Clarify the procedures for malaria and stool testing, including why these were done and ensure results are reported.

Authors response: Procedure for malaria is explained in the methods. Details of stool examinations and STHIs is a subject of a subsequent sub study being written up.

- Justify the collection of anthropometric data (e.g., weight) and blood samples state how much was collected, how often, and include SOPs on sample handling and transport if done across sites.

Authors response: Thank you for this observation, we have updated Table 1 to include the mean and standard deviation of participants’ weight.

- Explain how questionnaire data were matched with lab results and whether any systems were used to prevent data errors.

Authors response: Thank you for this comment, participants were assigned a unique ID, which was used consistently across questionnaires and lab forms. We have updated this under study measurements in the methods section, line number 171

Results, clarity and consistency

- Account for the reduced sample size and report the response rate.

Authors response: This has been addressed.

- Resolve inconsistencies in income and breadwinner data and align variable categories across tables (e.g., education and marital status).

Authors response: This has been addressed.

- Clarify variable definitions such as “time at camp,” “soil eating,” and how diagnoses (e.g., gastritis, HPV/cancer) and deworming history were established.

Authors response: This has been addressed.

Discussions and limitations

- Compare findings with studies among similar populations (i.e., pregnant refugees).

Authors response: Thank you for this comment, we have included comparative data from studies among refugee pregnant women in Ethiopia, Rwanda and Somalia. Line number 242-243

- Explain unexpected findings (e.g., higher anaemia in educated women).

Thank you. This has been revised. We now suggest that educated women may face occupational stress, delayed ANC initiation, and urbanized dietary habits low in iron-rich traditional foods. This could explain the unexpected association. Line number 261-264

- Discuss limitations of RDT-based malaria diagnosis and the omission of key variables like haematinic use.

We have included additional limitations, lack of data on haematinic use, limited parasite detection using RDT, and potential recall bias. Line number 278-287

- Add a brief section on the strengths of the study.

Authors response: Thank you, this has been done. Line number 278

==============================

Please submit your revised manuscript by Jun 29 2025 11:59PM. If you will need more time than this to complete your revisions, please reply to this message or contact the journal office at [plosone@plos.org](mailto:plosone@plos.org). When you're ready to submit your revision, log on to https://www.editorialmanager.com/pone/ and select the 'Submissions Needing Revision' folder to locate your manuscript file.

Please include the following items when submitting your revised manuscript:

- A rebuttal letter that responds to each point raised by the academic editor and reviewer(s). You should upload this letter as a separate file labeled 'Response to Reviewers'.
- A marked-up copy of your manuscript that highlights changes made to the original version. You should upload this as a separate file labeled 'Revised Manuscript with Track Changes'.
- An unmarked version of your revised paper without tracked changes. You should upload this as a separate file labeled 'Manuscript'.

If you would like to make changes to your financial disclosure, please include your updated statement in your cover letter. Guidelines for resubmitting your figure files are available below the reviewer comments at the end of this letter.

If applicable, we recommend that you deposit your laboratory protocols in protocols.io to enhance the reproducibility of your results. Protocols.io assigns your protocol its own identifier (DOI) so that it can be cited independently in the future. For instructions see: <https://journals.plos.org/plosone/s/submission-guidelines#loc-laboratory-protocols>. Additionally, PLOS ONE offers an option for publishing peer-reviewed Lab Protocol articles, which describe protocols hosted on protocols.io. Read more information on sharing protocols at <https://plos.org/protocols?utm_medium=editorial-email&utm_source=authorletters&utm_campaign=protocols>.

We look forward to receiving your revised manuscript.

Kind regards,

Henry Komakech, MHSR, B.A
Academic Editor
PLOS ONE

Journal Requirements:

When submitting your revision, we need you to address these additional requirements.

1. Please ensure that your manuscript meets PLOS ONE's style requirements, including those for file naming. The PLOS ONE style templates can be found at

<https://journals.plos.org/plosone/s/file?id=wjVg/PLOSOne_formatting_sample_main_body.pdf> and

<https://journals.plos.org/plosone/s/file?id=ba62/PLOSOne_formatting_sample_title_authors_affiliations.pdf>

2. Please include a complete copy of PLOS’ questionnaire on inclusivity in global research in your revised manuscript. Our policy for research in this area aims to improve transparency in the reporting of research performed outside of researchers’ own country or community. The policy applies to researchers who have travelled to a different country to conduct research, research with Indigenous populations or their lands, and research on cultural artefacts. The questionnaire can also be requested at the journal’s discretion for any other submissions, even if these conditions are not met.  Please find more information on the policy and a link to download a blank copy of the questionnaire here: https://journals.plos.org/plosone/s/best-practices-in-research-reporting. Please upload a completed version of your questionnaire as Supporting Information when you resubmit your manuscript.

3. Your ethics statement should only appear in the Methods section of your manuscript. If your ethics statement is written in any section besides the Methods, please delete it from any other section.

4. Please include captions for your Supporting Information files at the end of your manuscript, and update any in-text citations to match accordingly. Please see our Supporting Information guidelines for more information: <http://journals.plos.org/plosone/s/supporting-information>.

Additional Editor Comments:

The manuscript would benefit from additional contextual details about the study setting and clearer descriptions of methods, including sampling procedures, data collection processes, and justification for measurements conducted. Please also ensure consistency between the methods and results sections by including findings for all procedures mentioned and clarifying any discrepancies in tables and terminology used.

The authors should clarify discrepancies and definitions in the tables, particularly regarding income sources, education, marital status, and newly introduced variables like ‘soil eating.’ Additionally, explanations should be provided for how certain health conditions and variables were assessed, and missing methodological details should be included to ensure alignment between the methods and results sections.

The authors should provide appropriate comparisons with anaemia prevalence among pregnant refugee women in similar contexts and offer a clear, logical explanation for the unexpected association between education and anaemia in their findings. Additionally, they should explain the absence of gestational age data, expand on the study’s limitations, and highlight its key strengths.


[Note: HTML markup is below. Please do not edit.]

Reviewers' comments:

Reviewer's Responses to Questions

**Comments to the Author**

1. Is the manuscript technically sound, and do the data support the conclusions?

The manuscript must describe a technically sound piece of scientific research with data that supports the conclusions. Experiments must have been conducted rigorously, with appropriate controls, replication, and sample sizes. The conclusions must be drawn appropriately based on the data presented.

Reviewer #1: Yes

Reviewer #2: Yes

2. Has the statistical analysis been performed appropriately and rigorously?

Reviewer #1: I Don't Know

Reviewer #2: Yes

3. Have the authors made all data underlying the findings in their manuscript fully available?

The [PLOS Data policy](http://www.plosone.org/static/policies.action#sharing) requires authors to make all data underlying the findings described in their manuscript fully available without restriction, with rare exception (please refer to the Data Availability Statement in the manuscript PDF file). The data should be provided as part of the manuscript or its supporting information, or deposited to a public repository. For example, in addition to summary statistics, the data points behind means, medians and variance measures should be available. If there are restrictions on publicly sharing data—e.g. participant privacy or use of data from a third party—those must be specified.

Reviewer #1: No

Reviewer #2: Yes

4. Is the manuscript presented in an intelligible fashion and written in standard English?

PLOS ONE does not copyedit accepted manuscripts, so the language in submitted articles must be clear, correct, and unambiguous. Any typographical or grammatical errors should be corrected at revision, so please note any specific errors here.

Reviewer #1: Yes

Reviewer #2: Yes

5. Review Comments to the Author

Please use the space provided to explain your answers to the questions above. You may also include additional comments for the author, including concerns about dual publication, research ethics, or publication ethics. (Please upload your review as an attachment if it exceeds 20,000 characters)

We thank the reviewers for the insightful comments and valuable suggestions that have improved the clarity and quality of our manuscript. Below are our detailed responses.

Reviewer #1: General comments
The study addresses a critical public health issue in a vulnerable population and provides valuable localized data. There are however some issues the needs to be addressed or clarified to improve the quality of the manuscript.
Specific comments and recommendations
Study settings: The manuscript will benefit from more information on the setting eg – the geopolitical structure of the country, how healthcare delivery is structured in the country, who are the refugees and where did they migrate from, how long have they settled there, socio-economic context, etc.
What about the provision of routine haematinics, is it not part of the services offered at the ANC?

Authors response: This has been addressed.

Line 116 – Please explain this process of ‘random’ selection of 3 facilities. How many facilities in all were available? Did you do simple, systematic, or cluster selection? Or were the 3 facilities rather purposively selected?

The facilities were purposively selected based on levels and access to labs.

Line 117 – there is no reflection of Ayiri HC III in Table 1

Authors response: This has been revised. It is the 1^st^ row.

Line 135: In order to avoid confusion with qualitative interviews, I would suggest authors rephrase the use of the term “face-to-face interview” to ‘survey’ or a more unambiguous term.

Thank you for the suggestion, this has been done. Line number 135; *Data were collected through surveys administered by trained research assistants on information on maternal characteristics such as age, gravidity, education level, occupation, marital status, HIV status, and the number of ANC visits in the current pregnancy.*

Anthropometry: Measurement of weight is mentioned. Why was it done? There was no reflection of this in the results.

Thank you for this observation, we have updated Table 1 to include the mean and standard deviation of participants’ weight.

Malaria test: Blood film microscopic examinations were done along with parasite density. There was no reflection of this in the results

Authors response: This has been addressed. We have included Blood smear results

Line 185 – There was no mention of stool analysis in the methods

Authors response: we have removed this.

Results
Line 191 – Please state the reason(s) why the final sample was less than the calculated sample size i.e. why were the others excluded from the final analysis

Thank you for your comment, we were able to enroll 304 participants due to logistic issues within the settelemnt during the study. This has been updated in the methods section.

Table 1:
Please rephrase ‘Mother’s age’ to simple ‘Age’

Thank you, this has been revised accordingly. Line number: 203, Table 1

Source of income – Please clarify and reconcile the fact that 261 had no source of income, yet everyone had ‘estimated monthly income’. Furthermore, 267 were the bread-winners despite the majority not having a source of income?

Authors response: We agree this was a difficult question since most relied on reliefs from OPM and partners. However, we probed as much as possible and we have reported what the participants were able to tell us.

“Time at the camp’ – Does this mean that the refugees got to the area at different times? This underscores the earlier comment regarding more background information about the setting

Authors response: Yes, they came at different times
Table 2:
Gastritis/Ulcer – how were these determined? Please include in the methods
“HPV infection or cancer” – how were these diagnoses determined?
“Dewormed in last 6 months” – Please clarify how this was determined, and include the definition in the relevant section of the methods

Authors response: This were self-report and refelected now in the medthods.

Table 3:
Highest education level: please clarify those described as ‘none’, are they the same as ‘informal’ on Table 1?

Thank you, this has been revised.

Marital status: Pls clarify the components of the ‘single’ marital group, did this include those who were described as ‘separated’ on Table 1
‘Soil eating…’ is appearing for the first time here in a multivariable analysis?

Under Table 3, the “single” category includes those widowed, or separated. We have clarified this in the Table 3. Soil eating has now been included in the descriptive analysis in Table 2.

Discussion
Lines 251-254: You should rather proffer possible explanations for your own counter-intuitive finding instead of focusing on explaining the findings by others.

Thank you. This has been revised. We now suggest that educated women may face occupational stress, delayed ANC initiation, and urbanized dietary habits low in iron-rich traditional foods. This could explain the unexpected association. Line number 261-264

Limitations: The use of haematinics (yes or no) is a critical confounding variable not mentioned in the manuscript. Was this left out of the data collected? It appears this potentially compromised the validity of the findings.
The authors should also discuss the limitations associated with dependence on RDT for malaria diagnosis as presented in the results.

Thank you for your comment. We did not assess haematinic use, and we acknowledge this as a limitation that may have confounded results and have included it amongst our study limitations.

Reviewer #2: This study presents an investigation into the prevalence and severity of anaemia among pregnant refugee women settled in the West Nile region of Uganda, addressing a significant public health concern in a vulnerable population. The manuscript is clearly written, methodologically sound, and the findings are well presented. Nonetheless, several important areas require clarification and refinement. I have provided some constructive comments to improve the manuscript. Thank you

Abstract and Background

1. They are precise and well-written.

Methods

2. How did you randomly select the three facilities included in the study?

Authors response: Through lottery methods

3. For the reader's context, could you provide more details about which areas of the Adjumani district have refugees and how many facilities, among which you chose, serve them?

Authors response: This has been updated

4. Explain how the sample size was distributed among the three facilities.

Proportionately.

5. Line 138: Provide references to the studies alluded to.

Authors response: Thank you, this has been done. Line number 127

6. I note that complete blood count tests were all conducted at one facility (Mungula HCIV laboratory). Did you transport samples from other sites to this laboratory? If so, please provide a summary of the SOPs you followed (i.e vacutainers in which blood was collected, sample labelling, storage and transportation of the sample to the laboratory, time from collection to running the test etc). Please also attach the detailed SOPs used as a supplement file.

This has been updated. SOP attached.

7. You collected 4 mLs of CBC and another 4 mLs for malaria testing, a total of 8 mLs? According to your write-up, it seems you bled the patient twice. Is it so? Include a blood sampling section in the methods and clearly explain the procedures related to this.

Authors response: This has been revised and updated.

8. How did you synchronize patient records from the questionnaires to the test results from the laboratory? Did you use any system, was it pre-programmed to prevent mix-up of data. Please provide these details under your data collection procedures.

Authors response: Thank you for this comment, participants were assigned a unique ID, which was used consistently across questionnaires and lab forms. We have updated this under study measurements in the methods section, line number 171

9. You mention that patients were provided results of stool analysis, but that has not been elaborated in the study procedures. Please provide it, and what was the stool analysis performed for?

Authors response: This was an oversight, stool analysis was for a separate substudy.

Results

10. Line 191: Note the response rate of your study

Thank you, we have highlighted the response rate accordingly. Line number

11. In the study procedures, you performed microscopy and quantification of malaria parasites for participants, but none of this was reported in the results.

Authors response: We have reported this now.

Discussion

12. Line 233-237: From the references, I note that the studies from Tanzania, Ethiopia, India, Sudan and Ghana are not explicitly among refugee pregnant women, unless otherwise. You need to do some comparisons with the prevalence of pregnant refugee women elsewhere.

Thank you for this comment, we have included comparative data from studies among refugee pregnant women in Ethiopia, Rwanda and Somalia. Line number 242-243

13. Line 246-254: I found no explanation for why educated women in this study were likely to have anaemia, contrary to many other studies, as you noted. You instead gave a reason why educated women may have a lower prevalence of anaemia, as reported in different studies. Please provide a logical explanation for your finding.

Thank you, this has been revised accordingly. Line number: 261-264

14. Why were gestational ages not collected despite having access to the patients and their ANC card records as well?

Authors response: Thank you for this, however, we missed this on our dataset and acknolwdge it as a limitation.

15. Limitations of this study have not been exhaustively stated.

We have included additional limitations, lack of data on haematinic use, limited parasite detection using RDT, and potential recall bias. Line number 278-287

16. Please also state the strengths of your study.

Thank you, this has been done. Line number 278
